# An analysis of body proportions in children with CHARGE syndrome using photogrammetric anthropometry

**DOI:** 10.1002/ajmg.a.61215

**Published:** 2019-05-27

**Authors:** Bas Penders, Dieuwerke R. Dijk, Gianni Bocca, Luc J. I. Zimmermann, Conny M. A. van Ravenswaaij‐Arts, Willem‐Jan M. Gerver

**Affiliations:** ^1^ Department of Pediatrics Maastricht University Medical Center Maastricht The Netherlands; ^2^ Department of Genetics University of Groningen, University Medical Center Groningen Groningen The Netherlands; ^3^ Department of Pediatrics University of Groningen, University Medical Center Groningen Groningen The Netherlands

**Keywords:** body proportions, CHARGE syndrome, growth, photogrammetric anthropometry

## Abstract

**Background:**

Growth retardation is one of the main hallmarks of CHARGE syndrome (CS), yet little is known about the body proportions of these children. Knowledge of body proportions in CS may contribute to a better characterization of this syndrome. This knowledge is important when considering starting growth‐stimulating therapy.

**Methods:**

For this cross‐sectional study, we selected 32 children with CS and a CHD7 mutation at the Dutch CHARGE Family Day in 2016 or 2017 and the International CHARGE conference in Orlando, Florida, in 2017. We used photogrammetric anthropometry—a measurement method based on digital photographs—to determine various body proportions. We compared these to measurements in 21 normally proportioned children with growth hormone deficiency, using independent‐samples *t* test, Mann–Whitney *U* test, or chi‐square test as appropriate.

**Results:**

Children with CS appear to have a shorter trunk in proportion to their height, head length, and arm length. Children with CS also had smaller feet proportional to tibia length compared to controls. The change of body proportions with age was similar in children with CS and controls.

**Conclusion:**

Body proportions in children with CS are significantly different from those of normally proportioned controls, but a similar change of body proportions with age was noted for both groups.

## 1 INTRODUCTION

CHARGE syndrome (CS; OMIM 214800) is a rare disorder characterized by multiple anomalies. In 1981, Pagon introduced the acronym CHARGE, which summarizes some of the most prominent features present in the syndrome: coloboma of the eye, heart defects, atresia of the choanae, retardation of growth and/or development, genital hypoplasia, and ear and hearing abnormalities (Lalani et al., 2012; Pagon & Graham Jr., 1981). The occurrence and extent of these anomalies can be different among patients. The diagnostic criteria for CS were introduced by Blake and Verloes, using these and other frequent characteristics of CS such as aplasia of the semicircular canals and cranial nerve dysfunction (Blake et al., 1998; Verloes, 2005). In 2004, mutations in the *CHD7* gene (OMIM 608892) were identified to be responsible for the CHARGE phenotype (Vissers et al., 2004). Since then, many different mutations scattered throughout the *CHD7* gene have been found, and these are detected in up to 79% of patients with CS (Legendre et al., 2017). At present, the diagnosis of CS includes the results of genetic testing (Hale, Niederriter, Green, & Martin, 2016). The reported incidence of CS varies widely. In a Dutch cohort, the incidence was estimated to be 1/15,000 to 1/17,000 live births (Janssen et al., 2012).

Although growth retardation is one of the main characteristics of CS, little is known about the specific growth pattern in these children. Typically, children with CS have normal or slightly decreased birth weight and length. Within the first 3 months after birth, their growth rate decreases and patients with CS begin to show significantly lower weight and height than a reference population (Asakura et al., 2008; Blake, Kirk, & Ur, 1993; Dörr, Madeja, & Junghans, 2015; Legendre et al., 2017; Pinto et al., 2005). Some studies report catch‐up growth in the subsequent years of life, but height remains significantly below average in both boys and girls with CS (Blake et al., 1993; Dörr et al., 2015; Pinto et al., 2005; Shoji et al., 2014). The exact cause of this growth retardation remains unclear. A number of factors that might contribute to growth retardation have been suggested, including feeding difficulties, cardiac malformations, and endocrinological problems such as growth hormone deficiency and hypothyroidism. Of these, cardiac malformations and feeding difficulties are highly prevalent in CS (Blake & Hudson, 2017; Legendre et al., 2017), while growth hormone deficiency and hypothyroidism are found in a minority of patients. Prevalences of growth hormone deficiency varied between 12 and 34% in different studies. (Asakura et al., 2008; Legendre et al., 2017; Pinto et al., 2005). For hypothyroidism, prevalences between 0 and 16% were reported. (Asakura et al., 2008; Dörr et al., 2015; Legendre et al., 2017; Pinto et al., 2005; Shoji et al., 2014). In addition to reduced prepubertal growth, 75% of girls and 82% of boys with CS have gonadotropin deficiency and do not achieve spontaneous puberty (Bergman, Bocca, Hoefsloot, Meiners, & van Ravenswaaij‐Arts, 2011). Children with CS and hypogonadotropic hypogonadism do not have a pubertal growth spurt unless treated with sex hormones (Balasubramanian & Crowley, 2017). Despite these observations, the growth pattern and proportional development of children with CS remains largely unknown. More information about growth in children with CS would help physicians to monitor the development of these children.

Given how little is known about the growth pattern of children with CS, this cross‐sectional study was performed to assess body proportions in these children as compared to normally proportioned controls.

## 2 METHODS

This cross‐sectional study was designed and conducted in close collaboration by the Maastricht University Medical Center and the official Dutch expert center for CHARGE syndrome of the University Medical Center Groningen (UMCG).

### 2.1 Study groups

In total, 32 children with CS between 2 and 18 years of age were included in this study. Eleven children were included attending the Dutch CHARGE Family Day in 2016 or 2017, an annual event organized by the UMCG to provide parents of children with CS the opportunity to share experiences and to learn about new developments in research. In addition, 21 children with CS visiting the 13th International CHARGE Syndrome Conference in Orlando, Florida were included. Only children with a proven pathogenic variant of the *CHD7* gene were eligible for inclusion.

For the control group, 21 children between 2 and 14 years old visiting the outpatient clinic of Endocrinology and Growth of the Maastricht University Medical Center were included. These children were treated for growth hormone deficiency and were all proportioned normally according to the most recent Dutch reference values for body proportions (Gerver & Bruin, 2001). Before inclusion, all patients and/or their parents gave written informed consent. According to Dutch law, formal evaluation was waived by the institutional review board.

### 2.2 Measurements

**Figure 1:**
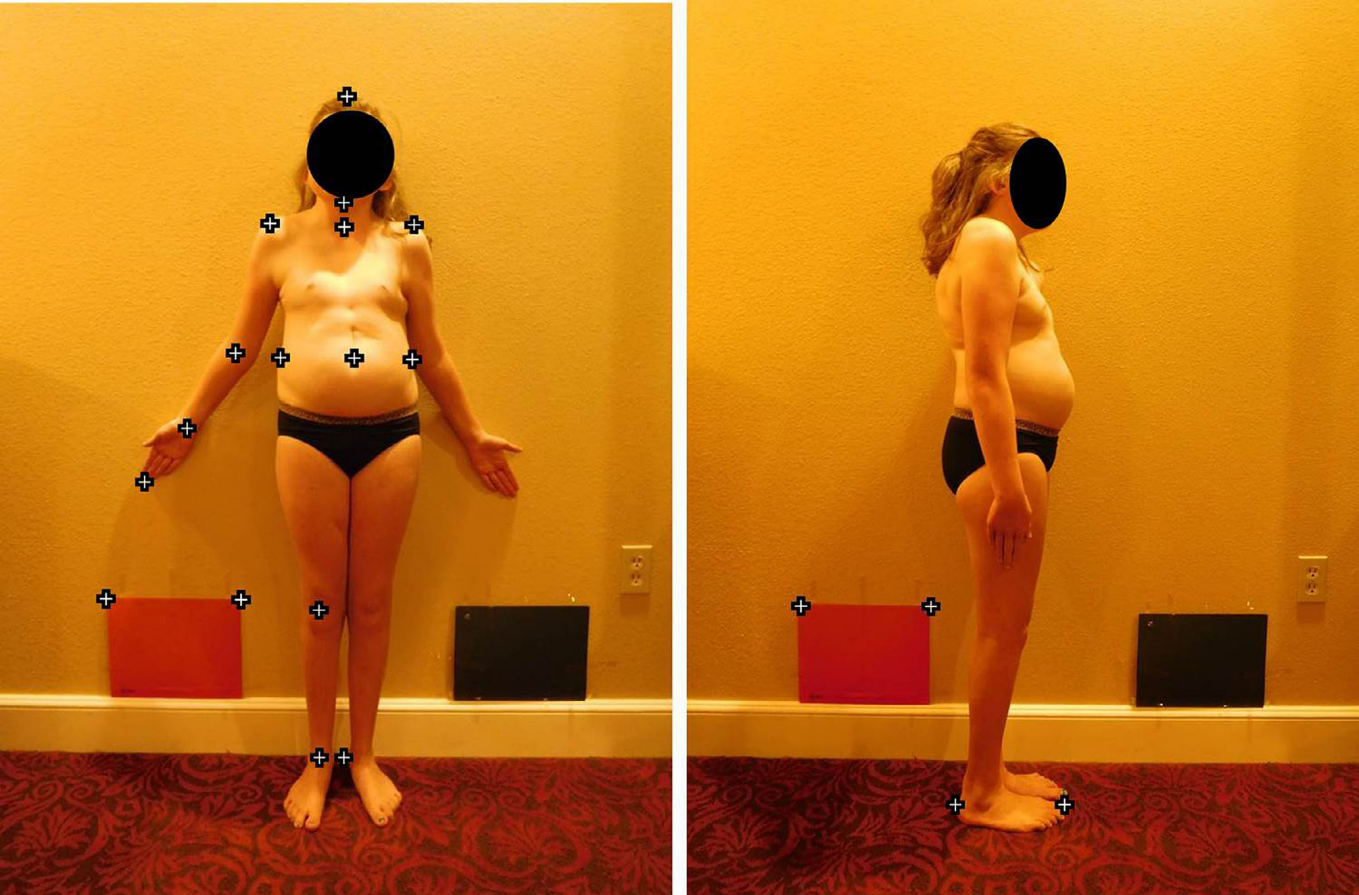
Position of the child with CHARGE syndrome. The reference measures are taped against the wall. The crosses represent the various reference points used to perform the anthropometric measurements on the photograph. Reproduced with permission from patient and parents [Color figure can be viewed at wileyonlinelibrary.com]

Digital photographs were taken of the children in underwear in frontal and lateral position, conforming to a photogrammetric method described previously (Penders, Brecheisen, Gerver, van Zonneveld, & Gerver, 2015). The children in the reference group were photographed at the Maastricht University outpatient clinic in standard anatomical position against a fixed backboard. For most children with CS, it is challenging to stand unaided in a standard anatomical position. Therefore, relatives accompanying the children were coached into aiding with the positioning process. Multiple photographs were taken to ensure that the various anthropometric measurements could be performed as accurately as possible. A reference measure of known size was used to compare the various measurements across photographs. Figure 1 shows photographs of a girl with CS in frontal and lateral positions, and the blue crosses represent the various measurements performed on the photographs. Body proportions were calculated from anthropometric measurements by selecting the anatomical reference points on these photographs using the photometry software *Paediatric Morphometrics* designed by our research group. This photometry technique is more patient‐friendly than taking elaborate manual measurements in children who are difficult to instruct and measure, and it was previously shown to provide consistent results with interobserver correlations ≥.96 (*p* ≤ .001) (Penders et al., 2015).

Various anthropometric measurements were determined on the photographs: height (H), head length (HeL), biacromial width (BiaW), biiliacal width (BiiW), upper arm length (UaL), lower arm length (LaL), hand length (HaL), tibia length (TiL), and foot length (FoL). These measurements were taken to conform with a standardized technique (Gerver & Bruin, 2001; Gripp, 2013). Additionally, trunk length (TrL) was determined on the photograph as the height difference between biacromial width and biiliacal width. Arm length (ArmL) was determined as the summation of upper arm length, lower arm length, and hand length.

### 2.3 Statistical analysis

All data were exported to IBM SPSS Statistics for Windows version 23.0 for statistical analysis. Shapiro–Wilk tests were performed for all measurements to test for normality. Comparison of different groups was done using independent samples *t* test, Mann–Whitney *U* test, or chi‐square test as appropriate.

## 3 RESULTS

### 3.1 Characteristics of the study participants

A total of 53 children were enrolled in this study (32 children with CS and 21 controls). There was a significant difference in gender distribution between the two groups, with no significant variation in age (Table 1). In total, 4 children with CS were known to have hypogonadotropic hypogonadism (2 Dutch children and 2 American), of which 3 were of pubertal age and have been receiving hormonal replacement therapy for 1–4 years. In all other children, hypogonadotropic hypogonadism was not yet diagnosed, these were all prepubertal and hence too young to start hormonal replacement therapy. One child with CS was known to have growth hormone deficiency and received adequate growth hormone therapy.

**Table 1:** Characteristics and measurements of the study participants

	Children with CHARGE	Controls	*p* value
*N*	32	21	
Male/female	11/21	16/5	.003*
Age ± SD	8.0 ± 4.67	10.2 ± 3.3	.069
Range	(2.6–18.7)	(2.6–14.8)	
HeL/H	0.18 ± 0.02	0.17 ± 0.02	.050*
HeL/TrL	0.67 ± 0.07	0.55 ± 0.06	<.001*
BiaW/BiiW	1.18 ± 0.12	1.22 ± 0.07	.230
BiaW/H	0.22 (0.17–0.28)	0.23 (0.21–0.23)	.537
BiiW/H	0.19 (0.15–0.22)	0.18 (0.17–0.20)	.541
TrL/H	0.27 ± 0.02	0.30 ± 0.02	<.001*
ArmL/H	0.40 (0.35–0.45)	0.43 (0.39–0.45)	.012*
ArmL/TrL	1.51 ± 0.15	1.37 ± 0.11	<.001*
UaL/LaL	1.25 ± 0.20	1.21 ± 0.07	.340
UaL/TiL	0.85 ± 0.10	0.76 ± 0.05	<.001*
TiL/H	0.21 (0.17–0.23)	0.22 (0.20–0.25)	<.001*
TiL/TrL	0.76 ± 0.10	0.75 ± 0.06	.787
FoL/TiL	0.67 ± 0.06	0.70 ± 0.05	.047*

*Note*: Data presented as mean ± SD or as median (min–max).

Abbreviations: ArmL, arm length; BiaW, biacromial width; BiiW, biiliacal width; FoL, foot length; HeL, head length; H, height; LaL, lower arm length; TiL, tibia length; TrL, trunk length; and UaL, upper arm length.

*Significance *p* ≤ .05.

Several body ratios showed significant differences between the CS group and the control group (see Table 1). These are head length/height, head length/trunk length, trunk length/height, arm length/height, arm length/trunk length, upper arm length/tibia length, tibia length/height, and foot length/tibia length. Additionally, *t* tests were performed to investigate if these body ratios were influenced by gender, and no significant differences were found (data not shown).

**GRAPH 1:**
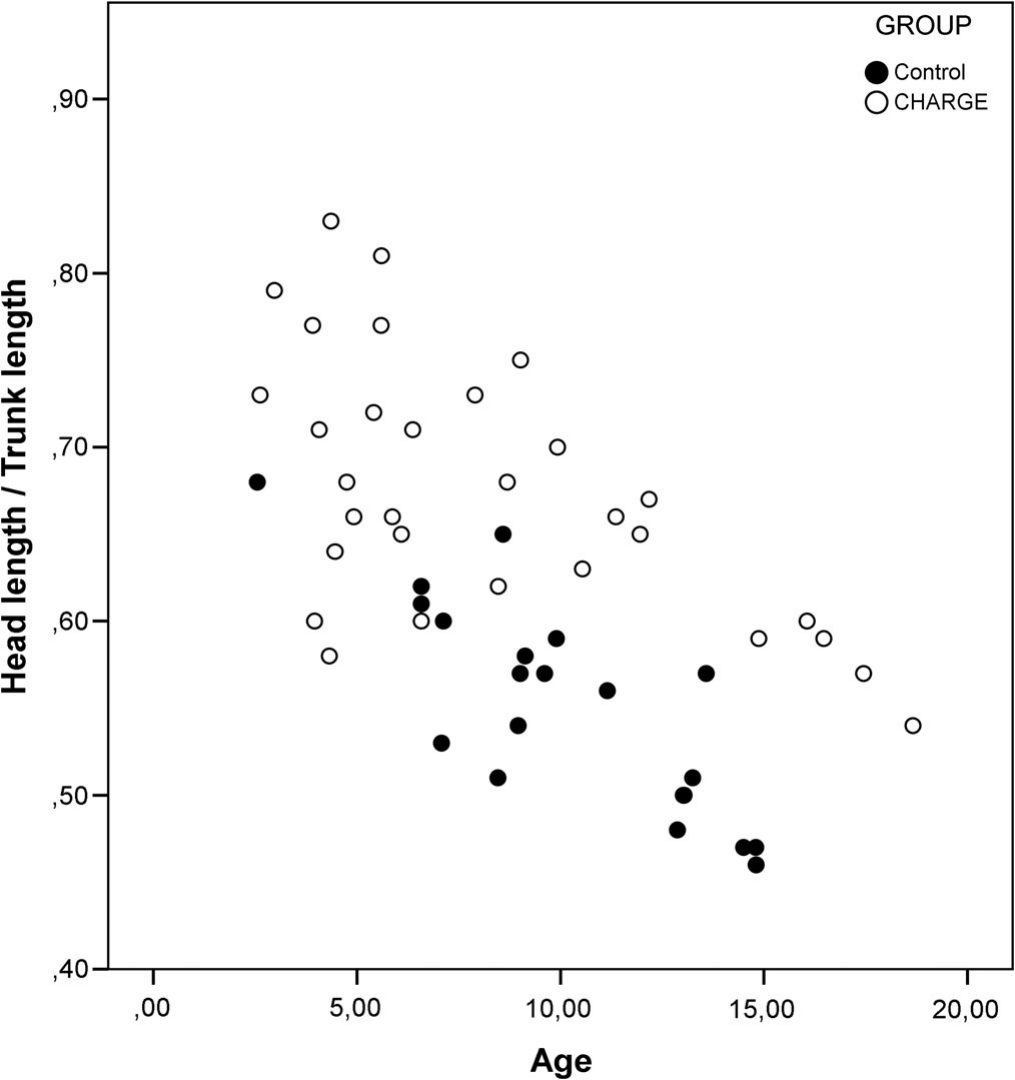
Head length/trunk length distribution for age

**GRAPH 2:**
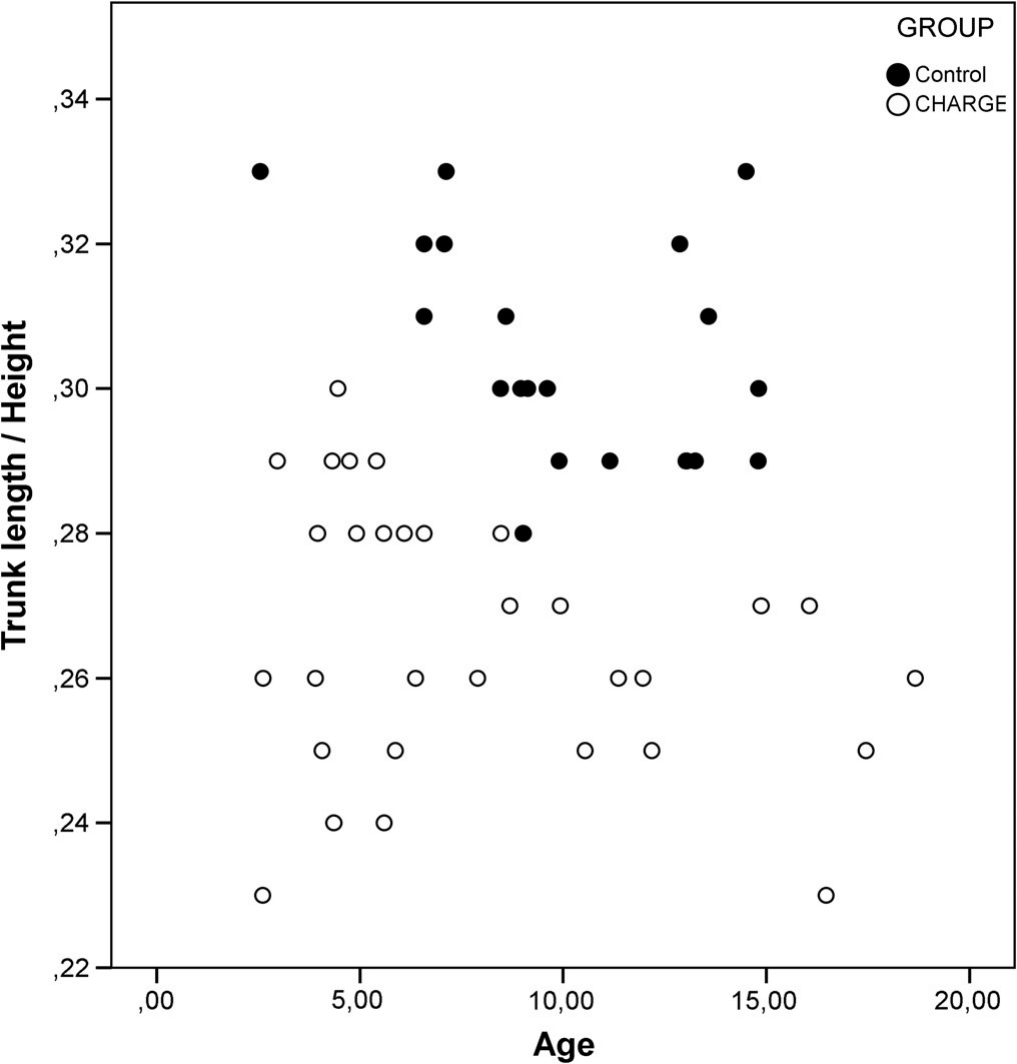
Trunk length/height distribution for age

**GRAPH 3:**
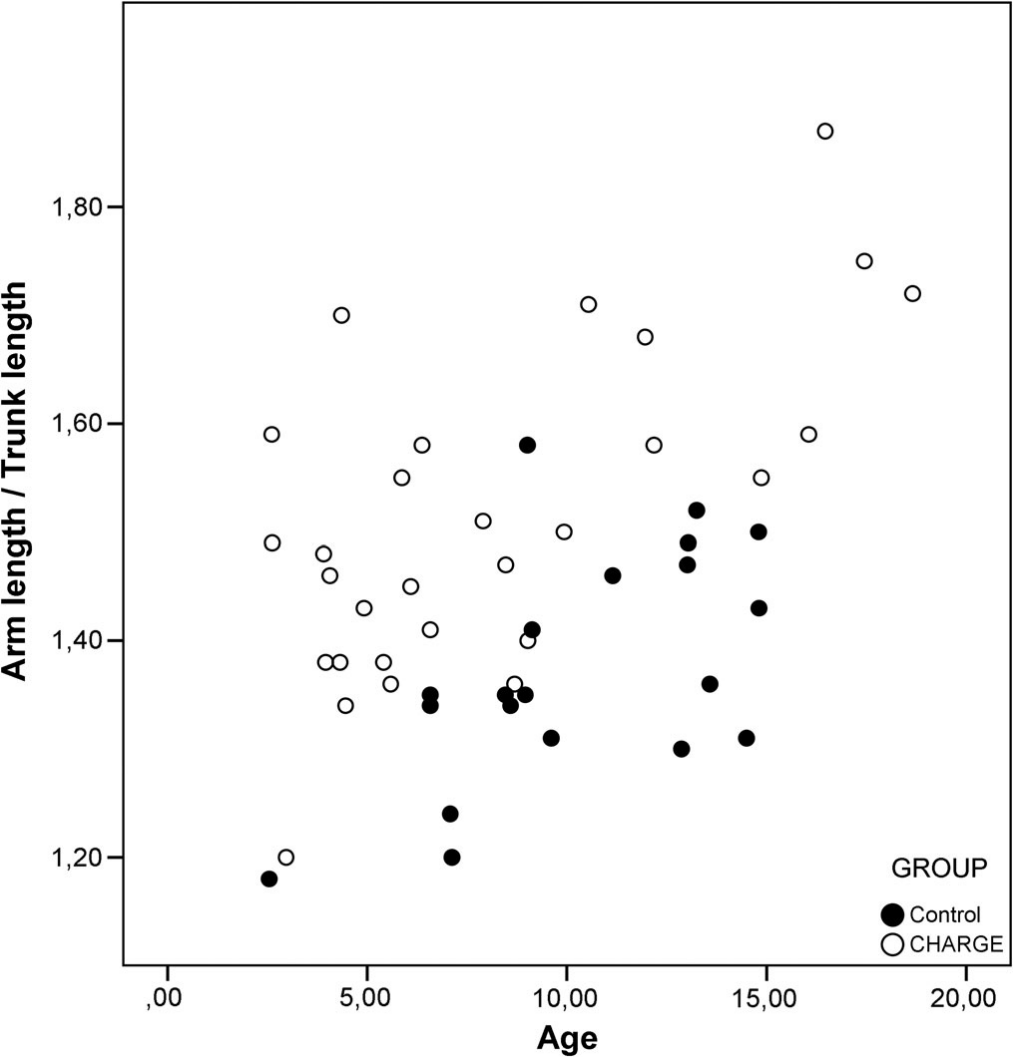
Arm length/trunk length distribution for age

Body proportions were analyzed in relation to age, and several body ratios showed an evident distinction in distribution. These distributions are presented in Graphs 1, 2, 3 and Data S1, Graphs 4–8, with black dots representing the control group and the white dots representing the CS group. The most illustrative graphs are presented in this article as Graphs 1, 2, 3. Graphs 4–8 are added as Data S1.

Graph 1 shows that children with CS have similar changes in body proportions with age to those of normally proportioned controls when looking at the ratio of head length to trunk length. With advancing age, the trunk becomes proportionally larger compared to the head. However, while the distribution pattern is similar, the proportional ratios are not. On average, children with CS have a shorter trunk in proportion to their head length compared to children in the control group. Graph 2 shows that the relatively shorter trunk is also observed when seen proportional to height (clustering of CS dots in the lower portion of Graph 2). As opposed to the HeL/TrL ratio (Graph 1), the TrL/H ratio (Graph 2) does not show a clear correlation with age, and this is true for both children with CS and the control group. Graph 3 shows that the change of proportion of arm length to trunk length with age in children with CS is similar to controls; however, children with CS appear to have longer arms on average in relation to their trunks (clustering of CS dots above controls in Graph 3).

## 4 DISCUSSION

### 4.1 Overview

The most significant difference found in this study is that children with CS appear to have a relatively shorter trunk than controls. This has been observed for trunk length in proportion to height, head length, and arm length. Of these body proportions, the relationship between trunk length and height does not show a clear correlation with age. This can be explained by the fact that the trunk length and height share a linear relationship. The total height increases at the same speed as the trunk length, which results in the proportional ratio remaining the same (Gerver & Bruin, 2001). As seen in Graph 2, the same proportional value of trunk length/height can be found across the whole age group. In addition, children with CS have relatively short feet. It is important to note that the relationship of the various body proportions to age appears to be similar in children with CS and normally proportioned controls. Therefore, we may conclude that the growth process of the body goes through the same proportional changes.

The relatively short trunk we found suggests that reduced spinal growth is an important part of the growth pattern in CS. This may be influenced by scoliosis, which is common in CS (Doyle & Blake, 2005). However, scoliosis typically progresses during growth, which would lead to a gradual decrease in trunk length in proportion to height, head length, and arm length. This does not correspond to our findings. Two of the Dutch children in the CS group were known to have slight scoliosis, which could be corrected by posture. Unfortunately, this information was not available for the American children.

Interestingly, the results of this study show some similarities to what has been found in children with Kabuki syndrome. In both syndromes, children appear to have shorter trunks proportional to their height, head length, and arm length (Penders, Schott, Gerver, & Stumpel, 2016). Previous studies have hinted at a possible association between these two chromatin remodeling disorders, and clinical overlap between CS and Kabuki syndrome has been described previously (Patel & Alkuraya, 2015; Verhagen, Oostdijk, Terwisscha van Scheltinga, Schalij‐Delfos, & van Bever, 2014). The clinical features shared between both syndromes include congenital heart defects, growth and/or developmental retardation, and urogenital malformations. The results of this study broaden this overlap to include a proportional distinction from normally proportioned controls.

### 4.2 Strengths

Apart from studies that described growth retardation in CS in general, this is the first study to investigate possible proportional anomalies in CS.

We used photogrammetric anthropometry, which allows for fast accumulation of data for measurement of body proportions. The strength of this method, opposed to classic anthropometry, is that several photographs can be combined to perform the measurements and no ideal pose needs to be found. This allows for a less invasive measurement technique, which proves to be more patient‐friendly than taking elaborate manual measurements and is also less frustrating for the observers. Additionally, the original data of the patient are preserved in the digital photograph, which makes retroactive measurements possible without needing to trouble the patient once more. In future longitudinal studies, this new technique will provide unique comparability potential in the monitoring of growth and development of individual patients, which is lost when simply comparing measurement values.

### 4.3 Weaknesses and considerations for future studies

This study makes use of a diversity of body proportions of which extensive reference values are not yet available. The first efforts in measurement of a control group of normally proportioned children have been made in a previous study, and these had to be used in this study to compare body proportions of children with CS to normally proportioned controls (Penders et al., 2015).

Of notice, the control group of our study consisted of children with growth hormone deficiency, and therefore by definition it is not a normal control group. However, children with growth hormone deficiency have normal body proportions, and we considered them to be a suitable control group with respect to the aims of our study.

In the present study, the distribution in gender is not the same across the observed groups. The CHARGE group contains a higher proportion of girls while the control group has more boys. To see if this difference would provide an undue representation of body ratios, *t* tests were performed, and we saw no significant differences based on gender. Children of over 15 years of age were underrepresented in the group with CS and were absent in the control group. This limits the possibility to draw conclusions about this age group. Since most children are prepubertal, and due to the small study population, no subdivision was possible according to pubertal stages. In this study population, a similar change in body proportions across age and gender was observed in both groups, which might be explained by the fact that most of the children were prepubertal. However, hypogonadotropic hypogonadism is common in CS and may influence the development of body proportions. In future studies, longitudinally following the growth and developmental process of patients with CS, it will be interesting to investigate the influence of delayed puberty or impact and timing of puberty induction on body proportions.

In addition, these data must be taken into consideration when evaluating the effect of growth stimulating therapies. When growth hormone treatment is started in children with CS, clinicians should be aware of the fact that, due to the relatively short trunk of these children, the effect of this therapy on final height may be less than expected. Moreover, it could be possible that the disproportions in children with CS, due to growth promoting therapy, will increase over time.

## 5 CONCLUSION

Children with CHARGE syndrome appear to have a shorter trunk in proportion to their height, head length, and arms. They also have, on average, smaller feet proportional to their tibia length compared to controls. No clear differences in change of body proportions with age are observed between children with CS and controls.

## CONFLICT OF INTEREST

The authors have no conflicts of interest to disclose.

## Supporting information


**Data S1**: Supporting information.

**Graph 4**: Head length/height distribution for age

**Graph 5**: Arm length/height distribution for age

**Graph 6**: Upper arm length/tibia length distribution for age

**Graph 7**: Tibia length/height distribution for age

**Graph 8** Foot length/tibia length distribution for age

## Data Availability

The data that support the findings of this study are available from the corresponding author upon reasonable request.
