# Supplementary material for: An analysis of body proportions in children with CHARGE syndrome using photogrammetric anthropometry
Source: Am J Med Genet A. 2019 May 27;179(8):1459–65. doi: 10.1002/ajmg.a.61215 (PMC6771509; doi:10.1002/ajmg.a.61215)
Supplement: Supplementary file 1 — Data S1: Supporting information. [file AJMG-179-1459-s001.docx]

**Supplementary materials**

Here, we present the additional graphs of the various body proportions which were found to be significantly different in children with CS as opposed to controls. The body proportions are presented for age, to allow for interpretation of the change in body proportions over time.

Graphs 4 and 5 show that the change in proportion of head length and arm length to height with age in children with CS is similar to controls. However, over time, arm length increases while head length decreases in proportion to height.

Graph 6 presents the relationship between upper arm length and tibia length, which does not show a clear distribution difference between children with CS and controls. The ratio of tibia length to height is presented in graph 7, which shows that, in younger children, the tibia length appears to be shorter relative to total height in children with CS compared to controls (clustering of CS dots in lower left portion of graph 7). In older children, this difference is less evident. Lastly, graph 8 reflects the relationship between foot length and tibia length, showing that the distribution across age is similar for both groups. However, children with CS have, on average, smaller feet proportional to their tibia length in comparison to controls.

**Graph 4.** Head length / Height distribution for age

**Graph 5.** Arm length / Height distribution for age

**Graph 6.** Upper arm length / Tibia length distribution for age

**Graph 7.** Tibia length / Height distribution for age

**Graph 8.** Foot length / Tibia length distribution for age
