# Supplementary material for: An analysis of body proportions in children with CHARGE syndrome using photogrammetric anthropometry
Source: Am J Med Genet A. 2019 May 27;179(8):1459–65. doi: 10.1002/ajmg.a.61215 (PMC6771509; doi:10.1002/ajmg.a.61215)
Supplement: Supplementary file 2 — Graph 4: Head length/height distribution for age [file AJMG-179-1459-s002.docx]

|  |
| --- |
| **Graph 4 \|** Head length/Height distribution for age |
