# Supplementary material for: An analysis of body proportions in children with CHARGE syndrome using photogrammetric anthropometry
Source: Am J Med Genet A. 2019 May 27;179(8):1459–65. doi: 10.1002/ajmg.a.61215 (PMC6771509; doi:10.1002/ajmg.a.61215)
Supplement: Supplementary file 5 — Graph 7: Tibia length/height distribution for age [file AJMG-179-1459-s005.docx]

**Graph 7 |** Tibial length/Height distribution for age
